# Subclinical Left Ventricular Systolic Dysfunction in Patients with Septic Shock Based on Sepsis-3 Definition: A Speckle-Tracking Echocardiography Study

**DOI:** 10.1155/2020/6098654

**Published:** 2020-09-21

**Authors:** Pham Dang Hai, Le Lan Phuong, Nguyen Manh Dung, Le Thi Viet Hoa, Do Van Quyen, Nguyen Xuan Chinh, Vu Duy Minh, Pham Nguyen Son

**Affiliations:** ^1^Intensive Care Unit, 108 Military Central Hospital, Ha Noi, Vietnam; ^2^Intensive Care Unit, Tam Anh General Hospital, Ha Noi, Vietnam; ^3^Ultrasound Department, 108 Military Central Hospital, Ha Noi, Vietnam; ^4^Department of Emergency, 108 Military Central Hospital, Ha Noi, Vietnam; ^5^Department of Cardiology, 108 Military Central Hospital, Ha Noi, Vietnam

## Abstract

**Introduction:**

Left ventricular dysfunction is quite common in septic shock. Speckle-tracking echocardiography (STE) is a novel, highly sensitive method for assessing left ventricular function, capable of detecting subclinical myocardial dysfunction, which is not identified with conventional echocardiography. We sought to evaluate subclinical left ventricular systolic function in patients with septic shock using speckle-tracking echocardiography.

**Methods:**

From May 2017 to December 2018, patients aged ≥18 years admitted to the intensive care unit with the diagnosis of sepsis and septic shock based on the sepsis-3 definition were included. Patients with other causes of cardiac dysfunction were excluded. Transthoracic echocardiography was performed for all the patients within 24 hours of diagnosis. Left ventricular systolic function was assessed using conventional echocardiography and speckle-tracking echocardiography.

**Results:**

Patients with septic shock (*n* = 90) (study group) and 37 matched patients with sepsis but no septic shock (control group) were included. Left ventricular ejection fraction (LVEF) by conventional echocardiography showed no significant difference between two groups (58.2 ± 9.9 vs. 58.6 ± 8.3, *p*=0.804). The global longitudinal strain (GLS) by STE was significantly reduced in patients with septic shock compared with that in the control (−14.6 ± 3.3 vs. −17.1 ± 3.3, *p* < 0.001). Based on the cutoff value of GLS ≥ −15% for the definition of subclinical left ventricular systolic dysfunction, this dysfunction was detected in 50 patients with septic shock (55.6%) and in 6 patients in the control group (16.2%) (*p* < 0.05).

**Conclusions:**

Speckle-tracking echocardiography can detect early subclinical left ventricular systolic dysfunction via the left ventricular global longitudinal strain compared with conventional echocardiographic parameters in patients with septic shock.

## 1. Background

Sepsis and septic shock are the main reasons for hospitalization and also the leading causes of death and disability in America and are associated with nearly US$17 billion in healthcare costs each year [1]. Cardiovascular system disorders are an essential component of septic shock, characterized by vasodilation and impaired cardiac function. The prevalence of cardiac dysfunction in patients with septic shock is approximately 60–70% [2, 3]. This disorder aggravates the condition of disease and increases the mortality rate.

Sepsis-induced myocardial dysfunction may include left ventricular systolic dysfunction, left ventricular diastolic dysfunction, and right ventricular dysfunction, alone or in combination.

Two-dimensional echocardiography is a noninvasive, low-cost imaging technique that allows both qualitative and quantitative evaluations of cardiac function in septic patients [4]. However, assessment of systolic function via left ventricular ejection fraction (LVEF) by conventional echocardiography depends on fluid resuscitation, preload, and afterload, and systolic function may be overestimated in the case of severe septic vasodilation [5]. Tissue Doppler imaging (TDI) has several limitations, including angle-dependent and less reproducible [6].

Speckle-tracking echocardiography (STE) is a new imaging technique that can overcome some of these limitations [7, 8]. It may allow a more in-depth analysis of the prevalence of left ventricular systolic dysfunction in septic shock and its early detection [9].

The purpose of this study is to assess left ventricular systolic function using speckle-tracking echocardiography in patients with septic shock. We hypothesize that speckle-tracking echocardiography is a sensitive tool in the early identification of left ventricular systolic dysfunction, which is not diagnosed by conventional echocardiography in septic shock patients.

## 2. Methods

### 2.1. Study Design and Setting

We performed a cross-sectional study in a twenty-five-bed intensive care unit of 108 Military Central Hospital from May 2017 to December 2018. The research protocol was approved by the institutional review board of 108 Military Central Hospital, Vietnam. Written informed consent was obtained from all participating patients or their legal representatives.

### 2.2. Study Population

All patients aged 18 years or older admitted for sepsis and septic shock based on the sepsis-3 definition [10] that developed within 24 hours before ICU admission were prospectively screened for eligibility. The study group included the patients with septic shock, and the control group consisted of age-matched, sex-matched, and cardiovascular risk-factor-matched patients with sepsis but did not develop septic shock.

Exclusion criteria included a documented ischemic heart disease at any point in the medical history, the presence of heart failure, moderate to severe valvular disease, valvular prosthesis, postthoracic operation, cardiac arrhythmia, postcardiopulmonary resuscitation status, poor echocardiographic image quality, and patients or their relatives who declined participation.

### 2.3. Data Collection

Baseline clinical variables within the first 24 hours after admission were collected from medical records including age, sex, comorbidities, hemodynamic parameters, vasopressor or inotropic dose, sequential organ failure assessment (SOFA) score [11], and acute physiology and chronic health evaluation (APACHE) II score [12]. The sources of infection were identified by fluid body cultures, including sputum, urine, blood, serous effusion, and cerebrospinal fluid.

### 2.4. Definitions

Sepsis and septic shock were defined according to the sepsis-3 definition [10]. Sepsis is defined as the syndrome of the presence of infection and a 2-point or greater increase in the SOFA score. Septic shock is identified as sepsis with persisting hypotension requiring vasopressors to maintain a mean arterial pressure ≥65 mmHg and a serum lactate level greater than 2 mmol/L despite adequate fluid resuscitation [10].

### 2.5. Two-Dimensional Transthoracic Echocardiography

Transthoracic echocardiography was performed for all the patients within 24 hours after the onset of septic shock or sepsis on the first day of ICU stay.

Echocardiographic exams were performed using Vivid S5 (GE Healthcare, USA) equipped with a 1.5–4 MHz phased array probe. All studies were performed by cardiologists with advanced training in echocardiography. All images and measurements were collected from standard views and digitally stored for offline analysis. Conventional echocardiographic measurements were obtained according to the guideline by the American Society of Echocardiography [13]. The following data were collected from the report: the left ventricular end-diastolic and systolic diameters (LVEDD and LVESD, respectively), left ventricular end-diastolic and systolic volumes, the left ventricular outflow tract (LVOT), velocity-time integral (VTI), left ventricular (LV) fractional shortening, and left ventricular ejection fraction (LVEF). LV fractional shortening was measured by M-mode echocardiography. The LVEF was measured using the biplane modified Simpson's method.

Cardiac output is calculated using velocity-time integration derived from pulse-wave Doppler echocardiography at the left ventricular outflow tract.

### 2.6. Two-Dimensional Speckle-Tracking Echocardiography

The LV global longitudinal strain (GLS) was calculated in the longitudinal three-chamber, two-chamber, and four-chamber views by 2D-speckle-tracking echocardiography with high-quality ECG gated images. The frame rate was set at between 50 and 90 frames/s, and a minimum of three cardiac cycles were obtained for each loop. The images were analyzed using software with the EchoPAC workstation (version 112, GE Healthcare, USA). The left ventricular endocardial border was manually traced in the end systole. Subsequently, software generates a speckle-tracking region-of-interest (ROI) to include the entire myocardium between the endocardium and the epicardium. The left ventricular was divided into 18 myocardial segments. Longitudinal strains for each segment were recorded and presented as a bull's eye. The strain values for all the segments are recorded and averaged to obtain the global longitudinal strain. GLS is presented as a percent change (%). Negative values of GLS indicate myocardial contraction. The predefined cutoff for subclinical left ventricular systolic dysfunction in patients with septic shock was defined by a GLS ≥ −15% (less negative than −15%) according to the previous studies [14–16].

### 2.7. Statistical Analysis

Statistical analysis was performed using SPSS version 20.0 software (SPSS, Inc., Chicago, IL, USA). The descriptive data were presented as mean value ± SD for continuous variables and as frequency (%) for categorical variables. Continuous variables were compared based on the Student's *t*-test or Mann–Whitney test. Categorical data were analyzed using the chi-squared test or a Fisher exact test. *p* value <0.05 was considered significant in two-tailed statistical tests.

## 3. Results

### 3.1. Patient Characteristics

**Figure 1:**
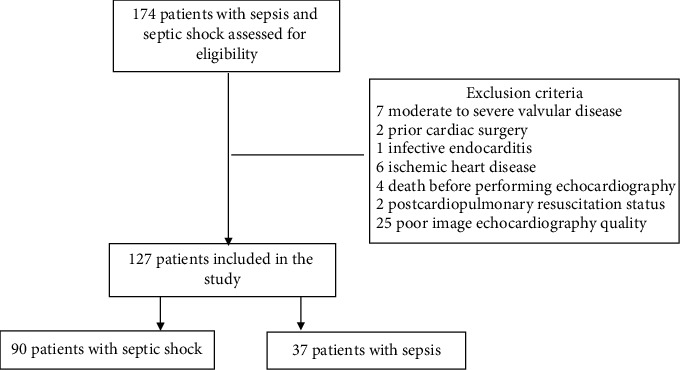
Inclusion/exclusion flowchart for study subjects.

Between May 2017 and December 2018, 174 consecutive adult patients were diagnosed with septic shock and sepsis, admitted to the ICU. Seven patients were excluded because of moderate to severe valvular disease, 2 because of prior cardiac surgery, 1 because of infective endocarditis, 6 because of ischemic heart disease, 2 because of postcardiopulmonary resuscitation status, 4 because of death before having echocardiography, and 25 because of insufficient image quality for STE analysis. The remaining 127 patients were eligible for assessment. All the patients were divided into two groups, including 90 patients with septic shock and 37 patients with sepsis as a control group. Details of the excluded patients are presented in Figure 1.

The baseline clinical characteristics of the analyzed patients are shown in Table 1.

The mean age was 68.8 ± 15.1 years and 64.1 ± 19.8 years in septic shock patients and control. There were no significant differences in age, sex, underlying diseases, or site of infection between the two groups. SOFA score and APACHE II score were significantly higher in patients with septic shock than the control (*p* < 0.001).

The proportion of patients with continuous renal replacement therapy and mechanical ventilation was significantly higher in the septic shock group compared with the control group (*p* < 0.001). In-hospital mortality rates in septic shock patients (43.3%) were significantly higher than those in the control (10.8%).

### 3.2. Hemodynamic Parameters

The septic shock group had a lower mean arterial pressure (70.4 ± 12.8 mmHg vs. 92.5 ± 11.7 mmHg, *p* < 0.001) and a higher heart rate (102.4 ± 18.7 bpm vs. 94.3 ± 18.9 bpm, *p*=0.028) compared with the control group. There were no significant differences in central venous pressure and cardiac output between the two groups.

### 3.3. Conventional Echocardiographic Parameters

Differences in LVEDV, LVEDS, LVEDD, and LVESD were not statistically significant between septic shock patients and the control. Left ventricular ejection fraction showed no significant difference between groups, *p* > 0.05 (Table 2).

### 3.4. Speckle-Tracking Echocardiographic Parameters

Septic shock patients showed significantly less negative values of longitudinal strain in apical 3-chamber view (−15.1 ± 3.7 vs. −17.1 ± 2.6, *p*=0.003), in apical 4-chamber view (−14.5 ± 3.3 vs. −17.0 ± 2.7), in apical 2-chamber view (−14.3 ± 3.9 vs. − 16.9 ± 2.8), and in global longitudinal strain (−14.6 ± 3.3 vs. −17.1 ± 3.3), *p* < 0.001 (Table 3). With a cutoff value of GLS ≥  −15% for the definition of subclinical left ventricular systolic dysfunction, 50 patients (55.6%) with septic shock had subclinical LV systolic dysfunction, while there were only 6 patients (16.2%) in the control group (*p* < 0.001).

## 4. Discussion

In the present study, we confirm the value of speckle-tracking echocardiography in evaluating left ventricular systolic dysfunction in septic shock. Our study demonstrated that the global longitudinal strain from STE was able to detect early impaired cardiac function, compared with LV ejection fraction from conventional echocardiography in septic shock. The septic shock group had a higher degree of myocardial dysfunction measured by left ventricular global longitudinal strain (GLS). Meanwhile, LVEF had no significant difference between the two groups of patients (*p* > 0.05). Our results are similar to the previous studies in which authors compared GLS by speckle-tracking echocardiography with LVEF measurement. Dalla et al. reported that septic shock patients have a lower GLS compared to patients with trauma and controls, despite no significant differences in LVEF [17]. Moreover, GLS in patients with severe sepsis and preserved LVEF was significantly impaired compared to critically ill, nonseptic trauma patients [17]. Similar results in longitudinal strain were reported by Ng et al. in patients with septic shock [9]. In the pediatric population, Basu et al. reported GLS decreased in septic patients compared with a control group, and LVEF had no significant difference in two groups [18].

The diagnosis of sepsis-induced myocardial dysfunction has been difficult because of the lack of the bedside diagnostic tool with high sensitivity and specificity. Although LVEF is used routinely for evaluating left ventricular systolic function, it is often affected by changes in preload and afterload, especially in septic shock [19, 20].

Global longitudinal strain (GLS) is a well-known index of LV systolic function and is sensitive to subendocardial fiber impairment [21]. So, it can be beneficial to monitor subclinical myocardial dysfunction in the early stages of several diseases such as hypertension, diabetes, ischemic heart diseases, and cardiotoxic chemotherapy; meanwhile, LVEF is still preserved [22–24].

Global longitudinal strain can be measured by multiple methods, including speckle-tracking echocardiography (STE), tissue Doppler imaging (TDI), and cardiovascular magnetic resonance (CMR) [21]. Speckle-tracking echocardiography is a newer, cost-effective, and reliable method for assessing LV systolic function, compared with tissue Doppler imaging and cardiovascular magnetic resonance [21]. Therefore, we used STE to detect the subclinical systolic dysfunction of LV in the present study.

Many previous studies have suggested that LV longitudinal systolic dysfunction should be defined when GLS ≥ −15% [14–16]. With this cutoff value, the proportion of left ventricular systolic dysfunction in the septic shock group in our study was relatively high (55.6%) and significantly higher than that in the control group (*p* < 0.05). Several studies have shown similar results with an impaired left ventricular systolic function in 30 to 60% of patients with septic shock [15, 17, 25].

This study revealed that although the LVEF was preserved, the GLS decreased in patients with septic shock. Thus, STE can detect impaired LV systolic function in early septic shock that may be missed by conventional echocardiography. The mechanisms underlying changes in the longitudinal strain with septic shock are not fully understood. Some hypotheses such as microvascular vasoconstriction in the subendocardial muscle layer [26] and altered coronary microvascular tone result in ischemic injury [27], myocardial depressant factor [28], proinflammatory mediators such as NF-*α* and IL-1*β*, and mitochondrial dysfunction [28]. Several factors affecting myocardial wall stress, such as preload and afterload, may affect longitudinal strain values [29–31]. However, Ho et al. demonstrated that strain did not depend on norepinephrine or phenylephrine in a rabbit model [30]. Several previous studies have shown that GLS is relatively independent of vasopressors and inotropes, volume loading, afterload, and extrinsic ventilator pressure, which most certainly affect cardiac mechanics [15, 19].

The early identification of sepsis-induced myocardial dysfunction might play an important role in the management of patients with septic shock by using *β*-blockers [32, 33].

This study had some limitations. Firstly, this was a single-center study with small sample size. Secondly, we did not evaluate the longitudinal strain during recovery from septic shock to determine whether longitudinal strain values are regressive baseline values. Thirdly, speckle-tracking echocardiography is a novel technique requiring adequate endocardial border identification, which may be challenging because of fluid resuscitation and mechanical ventilation. Fourthly, tachycardia is a common sign of sepsis and septic shock. However, a high heart rate may be a factor affecting global longitudinal strain measured by STE in sepsis and septic shock. Finally, our data were not compared to other modalities of quantifying ventricular function, such as cardiovascular magnetic resonance imaging.

## 5. Conclusion

In summary, speckle-tracking echocardiography can detect early subclinical left ventricular systolic dysfunction via the left ventricular global longitudinal strain, compared with conventional echocardiographic parameters in patients with septic shock. Speckle-tracking echocardiography may be a useful tool for early detection of septic cardiomyopathy.

## Figures and Tables

**Table 1:** Baseline characteristics and comparisons between patients with sepsis and those with septic shock.

	Septic shock (*n* = 90)	Sepsis (*n* = 37)	*p* value
Age, mean (years)	68.8 ± 15.1	64.1 ± 19.8	0.334
Male, *n* (%)	67 (74.4)	33 (89.2)	0.066
Comorbidities			
Hypertension, *n* (%)	37 (41.1)	21 (56.8)	0.109
Diabetes mellitus, *n* (%)	22 (24.4)	7 (18.9)	0.504
Stroke, *n* (%)	12 (13.3)	6 (16.2)	0.675
Chronic renal failure, *n* (%)	19 (21.1)	6 (16.2)	0.532
COPD, *n* (%)	2 (2.2)	3 (8.1)	0.123
Liver disease, *n* (%)	16 (17.8)	4 (10.8)	0.331
Source of infection			0.933
Abdominal	35 (38.9)	18 (48.7)	
Respiratory	43 (47.8)	16 (43.2)	
Kidney	5 (5.6)	1 (2.7)	
Skin	6 (6.6)	1 (2.7)	
Others	1 (1.1)	1 (2.7)	
Bacteremia	32 (35.6)	8 (21.6)	0.126
Gram-positive	6 (18.8)	3 (37.5)	
Gram-negative	21 (65.6)	4 (50)	
Others	5 (15.6)	1 (12.5)	
Pulse (beat/min)	102.4 ± 18.7	94.3 ± 18.9	0.028^*∗*^
MAP (mmHg)	70.4 ± 12.8	92.5 ± 11.7	<0.001^*∗*^
CVP (mmHg)	7.2 ± 2.7	6.6 ± 2.3	0.269
Norepinephrine (*μ*g/kg/min)	0.4 ± 0.4	—	—
CRRT, *n* (%)	51 (57.3)	6 (16.7)	<0.001^*∗*^
Mechanical ventilation, *n* (%)	80 (88.9)	23 (62.0)	<0.001^*∗*^
SOFA score	10.7 ± 3.3	4.5 ± 2.6	<0.001^*∗*^
APACHE II score	20.1 ± 7.9	12.7 ± 5.2	<0.001^*∗*^
ICU LOS, days	7.9 ± 7.2	5.6 ± 4.4	0.346
Hospital LOS, days	19.1 ± 15.6	19.1 ± 12.0	0.984
In-hospital mortality, *n* (%)	39 (43.3)	4 (10.8)	<0.001^*∗*^

Data are presented as means ± SD and number (*n*) of patients (%), as appropriate. APACHE II: acute physiology and chronic health evaluation, COPD: chronic obstructive pulmonary disease, CVP: central venous pressure, CRRT: continuous renal replacement therapy, ICU: intensive care unit, SOFA: sequential organ failure assessment, LOS: length of stay, and MAP: mean arterial pressure. ^*∗*^*p* < 0.05.

**Table 2:** Conventional echocardiographic variables.

	Septic shock (*n* = 90)	Sepsis (*n* = 37)	*p* value
LVEDV (mL)	99.2 ± 34.8	104.8 ± 23.6	0.246
LVESV (mL)	36.6 ± 16.5	38.9 ± 12.8	0.189
LVEDD (mm)	45.7 ± 7.0	47.1 ± 4.7	0.228
LVESD (mm)	29.8 ± 5.4	31.0 ± 4.3	0.158
LVEF (%)	58.2 ± 9.9	58.6 ± 8.3	0.804
FS (%)	34.7 ± 5.9	34.4 ± 5.1	0.535
LVOT	20.0 ± 1.7	20.5 ± 1.2	0.117
VTI (mm)	19.0 ± 4.0	19.2 ± 3.9	0.791
CO (L/min)	6.2 ± 1.8	6.0 ± 1.7	0.406

Data are presented as means ± SD. CO: cardiac output, LVEDV: left ventricular end-diastolic volume, LVESV: left ventricular end-systolic volume, LVESD: left ventricular end-systolic dimension, LVEDD: left ventricular end-diastolic dimension, LVEF: left ventricular ejection fraction, FS: fractional shortening, LOVT: left ventricular outflow tract, and VTI: velocity-time integral. ^*∗*^*p* < 0.05.

**Table 3:** Strain echocardiographic variables.

	Septic shock (*n* = 90)	Sepsis (*n* = 37)	*p* value
LS-A4C (%)	−14.5 ± 3.3	−17.0 ± 2.7	<0.001^*∗*^
LS-A2C (%)	−14.3 ± 3.9	−16.9 ± 2.8	<0.001^*∗*^
LS-A3C (%)	−15.1 ± 3.7	−17.1 ± 2.6	0.003^*∗*^
GLS (%)	−14.6 ± 3.3	−17.1 ± 3.3	<0.001^*∗*^
GLS ≥ –15%, *n* (%)	50 (55.6%)	6 (16.2%)	<0.001^*∗*^

Data are presented as means ± SD. GLS: global longitudinal strain by speckle-tracking echocardiography, LS: longitudinal strain, A3C: apical 3-chamber view, A4C: apical 4-chamber view, and A2C: apical 2-chamber view. ^*∗*^*p* < 0.05.

## Data Availability

The data used to support the findings of this study are available from the corresponding author upon request.

## Abbreviations

APACHE: Acute physiology and chronic health evaluation
A3C: Apical 3-chamber view
A4C: Apical 4-chamber view
A2C: Apical 2-chamber view
COPD: Chronic obstructive pulmonary disease
CO: Cardiac output
CRRT: Continuous renal replacement therapy
CVP: Central venous pressure
EF: Ejection fraction
FS: Fractional shortening
ICU: Intensive care unit
GLS: Global longitudinal strain
LS: Longitudinal strain
LV: Left ventricular
LVEDV: Left ventricular end-diastolic volume
LVESV: Left ventricular end-systolic volume
LVESD: Left ventricular end-systolic dimension
LVEDD: Left ventricular end-diastolic dimension
LOVT: Left ventricular outflow tract
SOFA: Sequential organ failure assessment
STE: Speckle-tracking echocardiography
TDI: Tissue Doppler imaging
VTI: Velocity-time integral.
